# Meta-analysis for the value of colchicine for the therapy of pericarditis and of postpericardiotomy syndrome

**DOI:** 10.1186/s12872-019-1190-4

**Published:** 2019-09-02

**Authors:** Leon L. Lutschinger, Angelos G. Rigopoulos, Peter Schlattmann, Marios Matiakis, Daniel Sedding, Christian Schulze, Michel Noutsias

**Affiliations:** 1 Department of Internal Medicine I, Division of Cardiology, Pneumology, Angiology and Intensive Medical Care, University Hospital Jena, Friedrich-Schiller-University Jena, Jena, Germany; 2 Mid-German Heart Center, Department of Internal Medicine III (KIM III), Division of Cardiology, Angiology and Intensive Medical Care, University Hospital Halle, Martin-Luther-University Halle-Wittenberg, Ernst-Grube-Strasse 40, D-06120 Halle (Saale), Germany; 3 Institute of Medical Statistics, Informatics and Data Science (IMSID), University Hospital Jena, Friedrich-Schiller University Jena, Jena, Germany

**Keywords:** Colchicine, Pericarditis, Post-pericardiotomy syndrome, Pericardial effusion, Therapy, Treatment

## Abstract

**Background:**

Colchicine has been used as anti-inflammatory agent in pericardial effusion (PE). We sought to perform a meta-analysis of randomized trials assessing the efficacy and safety of colchicine in patients with pericarditis or postpericardiotomy syndrome (PPS).

**Methods:**

In the systematic literature search following the PRISMA statement, 10 prospective randomized controlled studies with 1981 patients with an average follow-up duration of 13.6 months were identified.

**Results:**

Colchicine reduced the recurrence rate of pericarditis in patients with acute and recurrent pericarditis and reduced the incidence of PPS (RR: 0.57, 95% CI: 0.44–0.74). Additionally, the rate of rehospitalizations as well as the symptom duration after 72 h was significantly decreased in pericarditis (RR 0.33; 95% CI 0.18–0.60; and RR 0.43; 95% CI 0.34–0.54; respectively), but not in PPS. Treatment with colchicine was associated with significantly higher adverse event (AE) rates (RR 1.42; 95% CI 1.05–1.92), with gastrointestinal intolerance being the leading AE. The reported number needed to treat (NNT) for the prevention of recurrent pericarditis ranged between 3 and 5. The reported NNT for PPS prevention was 10, and the number needed to harm (NNH) was 12, respectively. Late colchicine administration > 7 days after heart surgery did not reduce postoperative PE.

**Conclusions:**

Our meta-analysis confirms that colchicine is efficacious and safe for prevention of recurrent pericarditis and PPS, while it reduces rehospitalizations and symptom duration in pericarditis. The clinical use of colchicine for the setting of PPS and postoperative PE after heart surgery should be investigated in further multicenter RCT.

## Background

Pericarditis and postpericardiotomy syndrome (PPS) are common diseases of the pericardium, which can be followed by serious and sometimes life-threatening complications, such as constrictive pericarditis or pericardial tamponade [1–3]. Typical clinical symptoms and signs include chest pain, pericardial friction rub, electrocardiographical abnormalities and pericardial effusion. Acute pericarditis is responsible for 5% of admissions to the emergency admissions due to chest pain [4]. It has a reported incidence of 27.7 cases /100,000 person-years [5] with a need for hospitalization of up to 3.32 cases/100000 person-years [6]. However, the true incidence may be higher, as many patients with pericarditis are not necessarily admitted to the hospital [7]. A common complication of pericarditis is the reappearance of symptoms 4–6 weeks after the acute event, which is denoted as incessant pericarditis [8]. A return of symptoms and/or clinical signs of pericarditis > 6 weeks after the acute event is defined recurrent pericarditis. Idiopathic pericarditis is complicated by recurrence in 15–50% of cases [5, 9, 10], while postpericardiotomy syndrome (PPS) occurs in 10–45% of cases after cardiac surgery [11].

In view of the high incidence of pericarditis recurrence and PPS there is a need for effective treatment in order to prevent recurrence and improve quality of life. While the administration of anti-inflammatory drugs, such as non-steroidal anti-inflammatory drugs (NSAID) or aspirin, has traditionally represented the mainstay of the standard therapy, including the abstinence from physical stress [12], colchicine has found in the last two decades its place in the treatment armamentarium and is being used as a standard treatment because of its anti-inflammatory action [13]. In the latest 2015 ESC Guidelines for the diagnosis and management of pericardial diseases, colchicine is recommended as first-line therapy for acute and recurrent pericarditis, as well as for the acute treatment of PPS [3].

In view of the lately increasing evidence, we carried out an updated meta-analysis of randomized trials to evaluate the efficacy and safety of colchicine in the prevention and treatment of pericarditis and of PPS.

## Methods

### Search strategy and selection criteria

Electronic searches were carried out using Medline (via PubMed), the Cochrane Library Embase and Web of Science following the PRISMA (Preferred Reporting Items for Systematic Reviews and Meta-Analyses) statement [14]. We combined the following key words / MeSH terms to identify the publications in the following query: “pericarditis OR pericardial effusion AND colchicine”. The literature search was conducted using EndNote Version X7.4 (Thomson Reuters), and after exclusion of duplicates resulted in 361 records. We applied the following inclusion criteria: studies investigating ≥10 patients with clinically pericarditis or post-pericardiotomy syndrome in which colchicine was compared against placebo. We excluded publications referring to only animal experiments or in vitro experiments, human studies on < 10 patients, case reports, congress reports, review articles, editorial letters, and publications written in languages other than English or German. The databases were searched by 2 independent reviewers (LLL and MN). The searches were reviewed by AGR. There were no discrepancies between the reviewers regarding the identified and classified literature. The retrieved studies and references were reviewed for relevance by using title and abstract. The full publications that met the above-mentioned criteria were retrieved and included in the analysis.

The primary aim of the study was to determine the efficacy of colchicine in the treatment and prevention of pericarditis and PPS. The secondary aim was the efficacy of colchicine in reducing the rehospitalization rate, symptom persistence after 72 h as well as the safety and adverse effects of colchicine treatment.

### Data extraction from the selected publications

Data regarding study characteristics, publication year, randomization mode, allocation concealment, blinding, intention-to-treat analysis, patient demographics and clinical characteristics, treatment indication and duration, colchicine dose, study and follow-up duration, clinical outcomes including pericarditis recurrence, re-hospitalizations, persistence of symptoms after 72 h and adverse effects were recorded.

### Assessment of study quality

Quality and risk of bias of the included studies was assessed using the Jadad scale [15]. This tool includes assessment of randomization, blinding of participants, and recording of dropouts and thus defines a Jadad score for each included study, whose value can be interpreted in terms of the probability of bias.

### Statistical analysis

Statistical analyses were performed using the software package R (The R Project for Statistical Computing; version 3.3.2) with the packages “meta” and “metafor”, which were employed for calculation of heterogeneity between the studies, Forest plots and Funnel plots [16]. Categorical variables were reported as percentages and continues variables as mean ± standard deviation (SD). A probability value of *p* < 0.05 was considered statistically significant. Risk ratios (RRs) with 95% confidence intervals (CIs) were used for all outcomes. Heterogeneity was assessed by means of the I^2^ statistic [17]. The risk of bias was assessed with the function “metabias” of the “meta” package of R and presented as a funnel plot.

## Results

### Included publications and patients

**Fig. 1:**
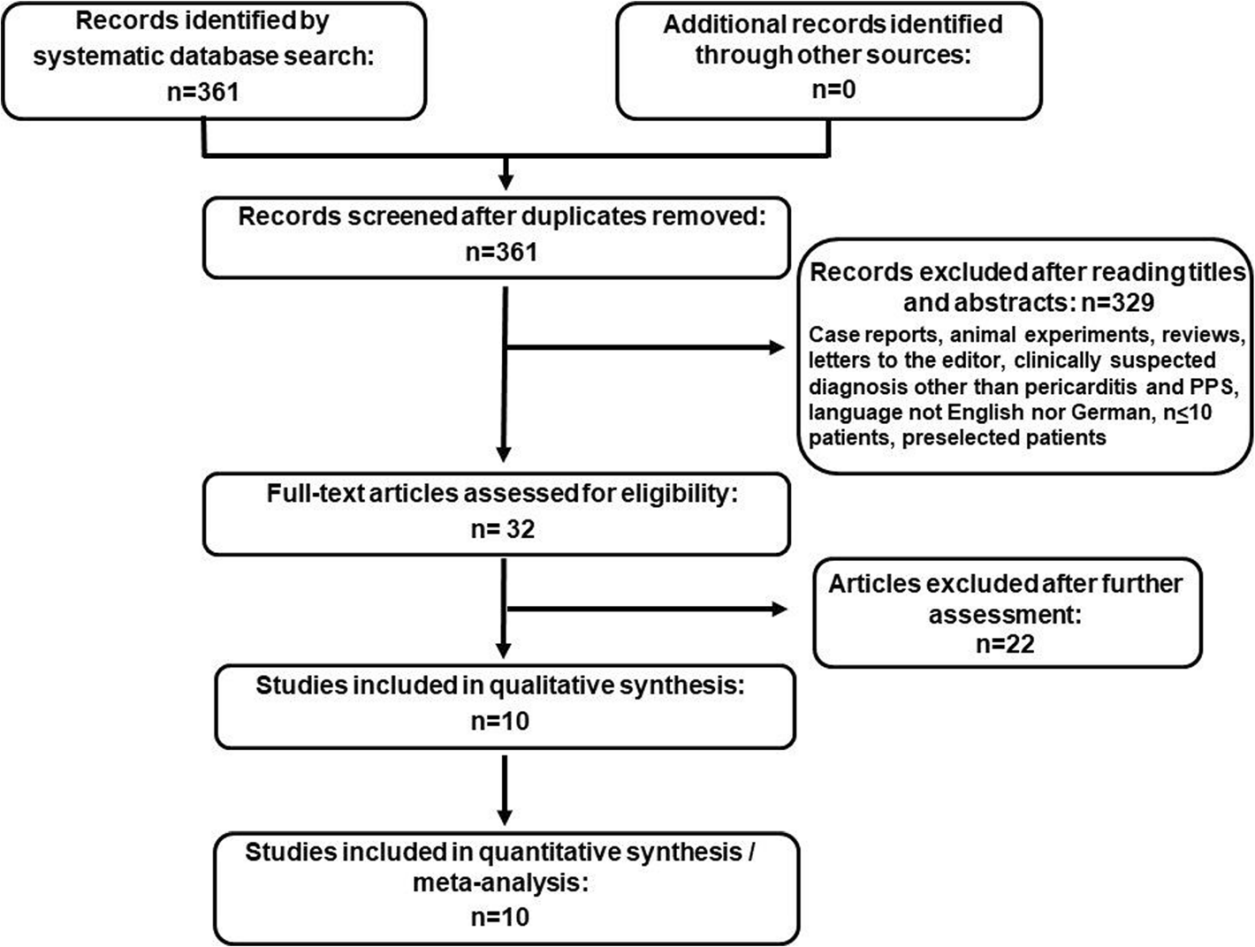
Flow chart for the selection of studies. The flow diagram represents the number of studies reviewed and included in the analysis

A total of 361 studies from 1977 until 2016 were retrieved in the original search (Fig. 1). After reviewing titles and abstracts for the inclusion and exclusion criteria, 329 were excluded as not relevant. Full text evaluation of the remaining 32 publications resulted to the selection of 10 randomized controlled trials (RCT) to be included in this meta-analysis. Eight of them were double blinded while the rest had an open-label design. Intention-to-treat analysis was included in eight studies while it was not mentioned in the rest two studies. Nine studies were multicenter while one was performed in one center. The follow-up time was different in each study and reached from one up to 24 months (mean follow-up: 13.6 months). Only five patients were lost to follow-up in one study [18]. No patients lost to follow-up were reported in the remaining studies.

The indication for colchicine therapy varied as follows: two studies included patients with the first acute pericarditis incident, three studies included patients with recurrent pericarditis, and the rest five studies described the action of colchicine in postpericardiotomy syndrome (PPS) and postoperative pericardial effusion.

The primary endpoint in the five studies of pericarditis was recurrence of pericarditis, while in the three PPS studies the incidence of PPS, and in the remaining two studies the reduction of postoperative pericardial fluid. Secondary endpoints were the rate of rehospitalization (*n* = 5 studies) as well as the symptom persistence after 72 h (n = 5 studies).

The characteristics of the 10 studies included in the meta-analysis are listed in Table 1**.** The 10 prospective studies from 2002 to 2015 included 1981 patients (mean age of 57.6 ± 7.3 years, 62% males). The 1000 control patients (57.9 ± 8.0 years) received as standard therapy NSAID (e.g. ibuprofen) or ASA, and only rarely corticosteroids (prednisone). Instead of colchicine, the control patients received placebo. The weight-adjusted doses of colchicine were in seven studies (0.5–1.0 mg), in two studies 1.0 mg and in the rest of the included studies 1.5 mg.

**Table 1:** Included studies and baseline characteristics of the patients

Study code ^a^	Publication, first author, study acronym	Year	Study design	Indication	Number of patients	Male gender (%)	Mean age (years)	Treatment duration (months)	Follow-up (months)
01	Finkelstein Y. et al. [19]	2002	Multicenter, randomized, double blind	PPS prevention	111	73	64.0	1	3
02	Imazio M. et al., CORE [20]	2005	Single center, randomized, open label	PER recurrence	84	35	53.8	6	20
03	Imazio M. et al., COPE [21]	2005	Multicenter, randomized, open label	PER first episode	120	45	56.9	3	24
04	Imazio M. et al., COPPS [22]	2010	Multicenter, randomized, double blind	PPS prevention	360	66	65.7	1	19
05	Imazio M. et al., CORP [23]	2011	Multicenter, randomized, double blind	PER recurrence	120	52.5	47.6	6	18
06	Imazio M. et al., ICAP [24]	2013	Multicenter, randomized, double blind	PER first episode	240	60	52.1	3	22
07	Imazio M. et al., COPPS-2 (11)	2014	Multicenter, randomized, double blind	PPS prevention	360	69	67.5	1	3,1
08	Imazio M. et al., CORP-2 (10)	2014	Multicenter, randomized, double blind	PER recurrence	240	50	48.8	6	20
09	Meurin P. et al., POPE-2 (21)	2015	Multicenter, randomized, double blind	PE post heart surgery	197	86	64.5	0.5	6
10	Izadi Amoli A. et al., [18]	2015	Single center, randomized, triple blind	PE post heart surgery	149	60	57.4	0.5	1

^a^study code of the publications included in the meta-analysis

*PE* pericardial effusion, *PER* pericarditis, *PPS* post-pericardiotomy syndrome

The duration, adverse effects (AE) of colchicine treatment, adherence and drug withdrawal (DW) are summarized in Table 2. The duration of colchicine treatment in pericarditis patients was initiated early after clinical diagnosis and randomization, and was maintained for 3–6 months. In contrast, the duration of colchicine treatment in patients undergoing heart surgery ranged between 14 days and 4 weeks, and was initiated 48–72 h before heart surgery [11], at the 3rd postoperative day [19, 22], at day 7–30 [25], or at 3 weeks after heart surgery [18] (Table 2).

**Table 2:** Duration, adverse effects, adherence and drug withdrawal of colchicine treatment

Study code^a^	Duration of colchicine treatment (and treatment start post heart surgery in PPS)	Adverse effects (AE) [%], non-adherence (NA) [%] and drug withdrawal (DW) [%]
01	1 month (start at 3rd postoperative day)	SAE: 0% AE: 14.1% ­ GI: 11.7% ­ AR: 0.6% ­ RF: 1.2% ­ PR: 0.6% NA: 10.4% DW: n.r.
02	6 months	SAE: 0% AE: 7% ­ GI: 7% NA: 0% DW: n.r.
03	3 months	SAE: 0% AE: 8.3% ­ GI: 8.3% NA: 0% DW: n.r.
04	1 month (start at 3rd postoperative day)	SAE: 0% AE: 8.9% ­ GI: 8.9% NA: n.r. DW: 11.7% ­ DW-AE: 8.9% ­ DW-OR: 2.8%
05	6 months	SAE: 0% AE: 11.7% ­ GI: 7% NA: n.r. DW: 8% ­ DW-AE: 7% ­ DW-OR: 1%
06	3 months	SAE: 0% AE: 11.7% ­ GI: 7% ­ HT: 1.7% ­ AP: 0.8% NA: < 5% DW: 11.7% ­ DW-AE: 11.7% DW-OR: 0%
07	1 month (start at 48–72 h before heart surgery)	SAE: 0% AE: 20% ­ GI: 14.4% ­ HT: 0.6% NA: < 5% DW: 21.7% ­ DW-AE: 20% ­ DW-OR: 1.7%
08	6 months	SAE: 0% AE: 11.7% ­ GI: 7.5% ­ HT: 2.5% ­ MT: 0.8% ­ AP: 0.8% NA: < 5% DW: 6.7% ­ DW-AE: 6.7% ­ DW-OR: 0%
09	14 days (start at 7–30 days after heart surgery)	SAE: 0% AE: 10.2% ­ GI: 9.2% ­ LP: 1% NA: 9.2% DW: 10.2% ­ DW-AE: 10.2%
10	14 days (start at 3 weeks after heart surgery)	SAE: 0% AE: n.r. NA: n.r. DW: n.r.

^a^study code of the publications included in the meta-analysis

*n.r.* value not referred in the publication, *SAE* serious adverse effects, *AE* adverse effects, *GI* gastrointestinal intolerance, *AR* allergic reaction, *RF* renal failure, *PR* pancreatitis, *HT* hepatotoxicity, *AP* alopecia, *LP* leucopenia, *NA* non-adherence, *DW* drug-withdrawal, *DW-AE* drug-withdrawal due to adverse effects, *DW-OR* drug-withdrawal due to other reasons (e.g. patient or medical decision)

**Fig. 2:**
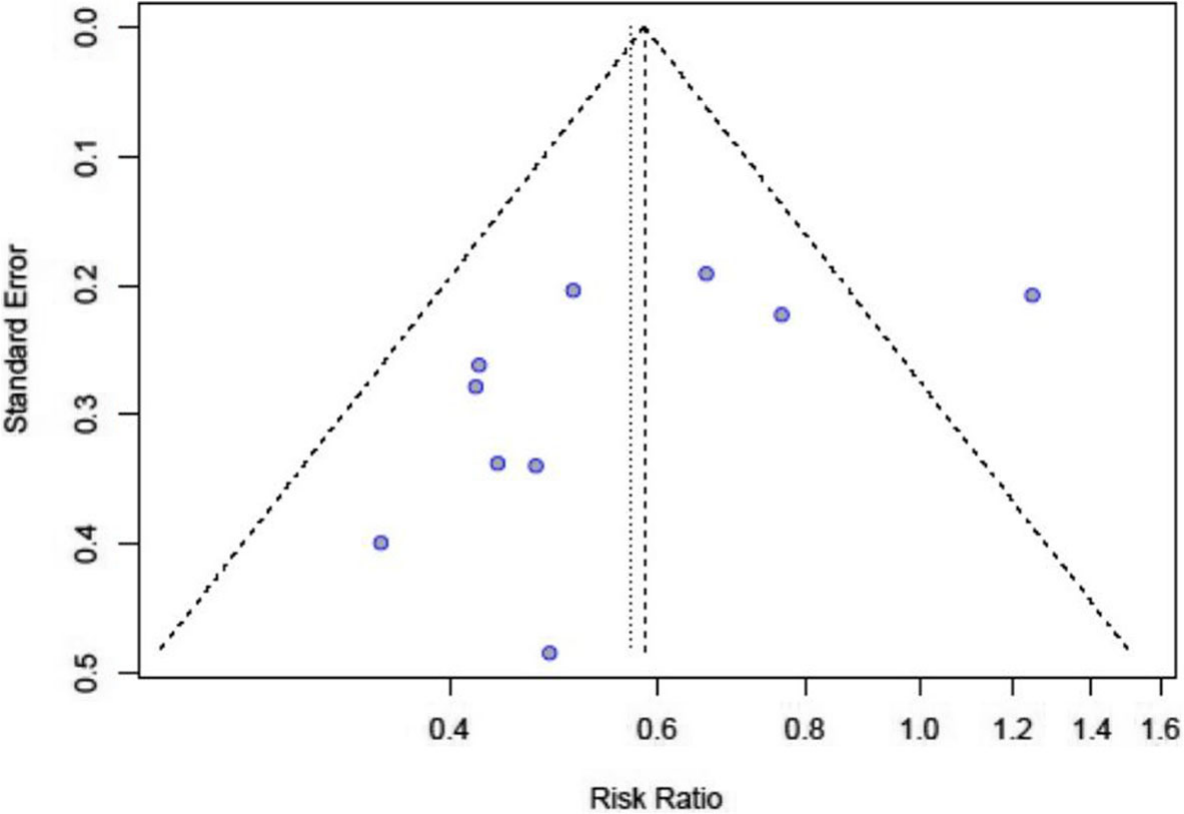
Funnel plot of the studies included in the meta-analysis. The individual studies are represented by blue dots and they distributed around the mean value, which is represented by the dotted vertical line. The risk ratio is depicted on the X axis opposite to the standard error on the Y axis

The assessment of the included studies according to the Jadad scale is presented in Table 3**.** The funnel plot of the studies included in the meta-analysis is shown in Fig. 2**.**

**Table 3:** Jadad score of the studies in the meta-analysis

Study code #	Randomization	Randomization appropriate	Blinding	Blinding appropriate	Dropouts	Total score
01	1	–	1	–	1	–
02	1	1	0		1	3
03	1	1	0		1	3
04	1	1	1	1	1	5
05	1	1	1	1	1	5
06	1	1	1	1	1	5
07	1	1	1	1	1	5
08	1	1	1	1	1	5
09	1	1	1	1	1	5
10	1	1	1	1	1	5
Total:						4.6

### Prevention of PE in PC and PPS

**Fig. 3:**
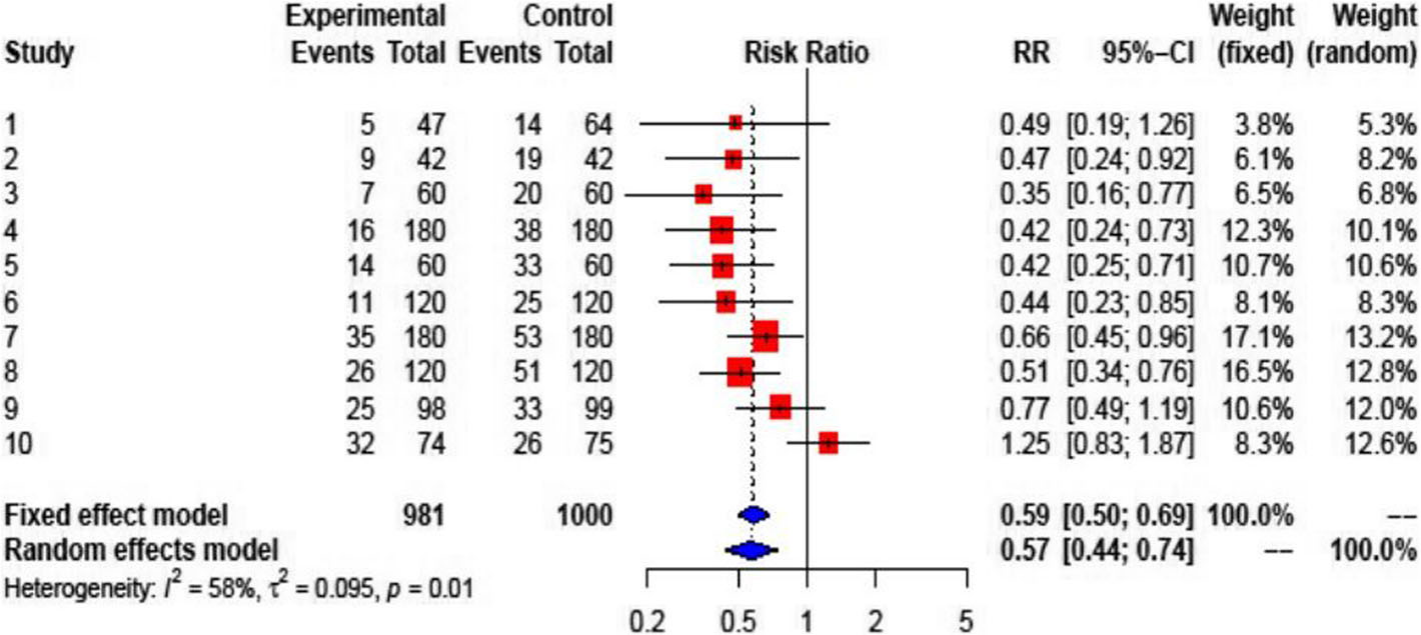
Forest plot graph for the meta-analysis on PE prevention or recurrent PE in pericarditis or post-operative patients by colchicine. The mean relative risk (RR) is represented with the blue diamond and the dotted vertical line. The red squares show the RR for the individual studies, the horizontal lines show the corresponding 95% confidence intervals. Experimental: study group treated with colchicine; Control: control group. Events: sum of events, Total: number of patients of the corresponding group, RR: relative risk

**Fig. 4:**
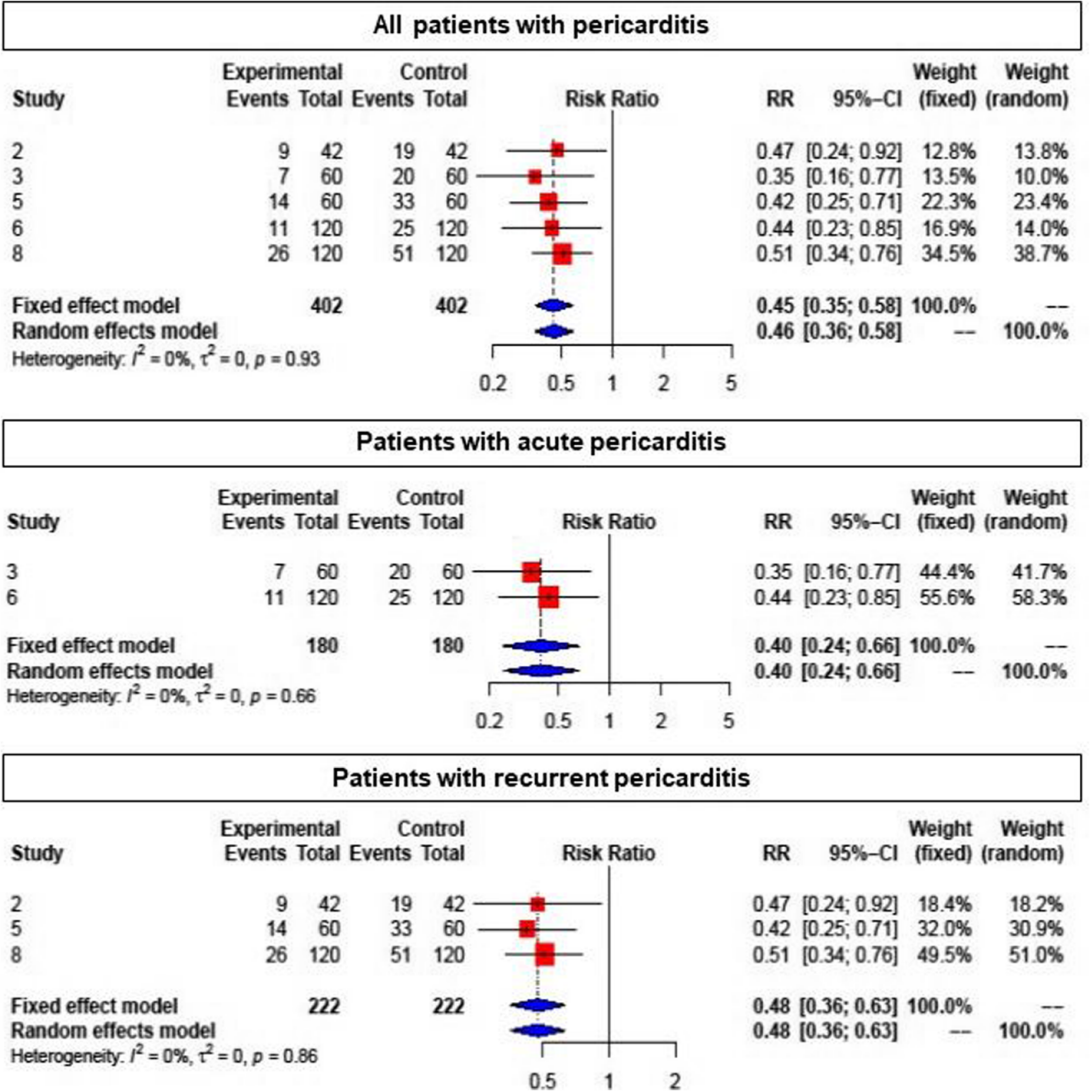
Forest plot graph for the meta-analysis of pericarditis recurrence under colchicine. Upper graph: all patients with pericarditis, middle graph: patients with acute pericarditis, bottom graph: patients with recurrent pericarditis. The mean relative risk (RR) is represented with the blue diamond and the dotted vertical line. The red squares show the RR for the individual studies, the horizontal lines show the corresponding 95% confidence intervals. Experimental: study group treated with colchicine; Control: control group. Events: sum of events, Total: number of patients of the corresponding group, RR: relative risk

**Fig. 5:**
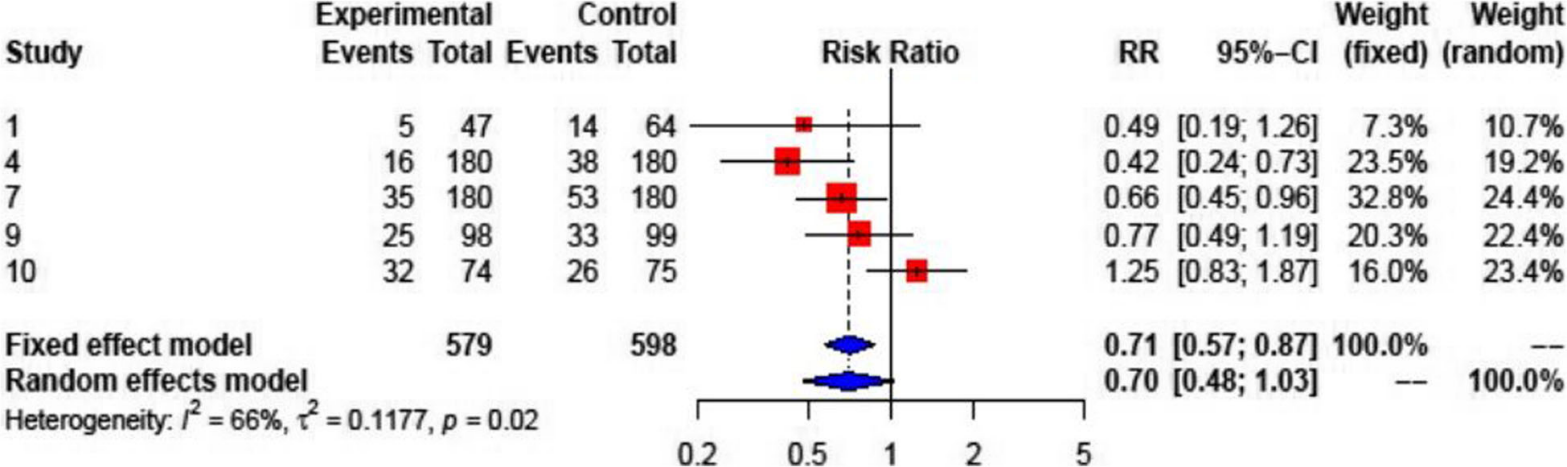
Forest plot graph for the meta-analysis of the incidence of Post-Pericardiotomy Syndrome (PPS). The mean relative risk (RR) is represented with the blue diamond and the dotted vertical line. The red squares show the RR for the individual studies, the horizontal lines show the corresponding 95% confidence intervals. Experimental: study group treated with colchicine; Control: control group. Events: sum of events, Total: number of patients of the corresponding group, RR: relative risk

Colchicine was shown to reduce the overall risk of PE in PC and recurrent pericarditis, and in PPS (all 10 studies) compared with placebo (RR: 0.57; 95% CI: 0.44–0.74 (Fig. 3). Due to the different populations studied, there was a considerable heterogeneity among studies (I^2^ = 58%, *p* = 0.01). In patients with pericarditis (5 studies), colchicine reduced the risk of recurrence (RR 0.46; 95% CI: 0.36–0.58) (Fig. 4, upper panel). Both patients with a first acute pericarditis (2 studies; RR: 0.40; 95% CI: 0.24–0.66) as well as patients with recurrent pericarditis (3 studies; RR: 0.48; 95% CI: 0.36–0.63 benefited from colchicine treatment (Fig. 4, middle and lower panel respectively). The reported number needed to treat (NNT) for the prevention of recurrent pericarditis was 3 and 5, respectively [10, 23]. For acute pericarditis, the reported NNT was 4 and 5, respectively [21, 24]. Colchicine did not prove superiority compared to placebo for prevention of PPS in patients after heart surgery (RR: 0.70; 95% CI: 0.48–1.03) (Fig. 5). One RCT for PPS prevention reported an NNT of 10, and a number needed to harm (NNH) of 12 [11].

### Rate of rehospitalization

**Fig. 6:**
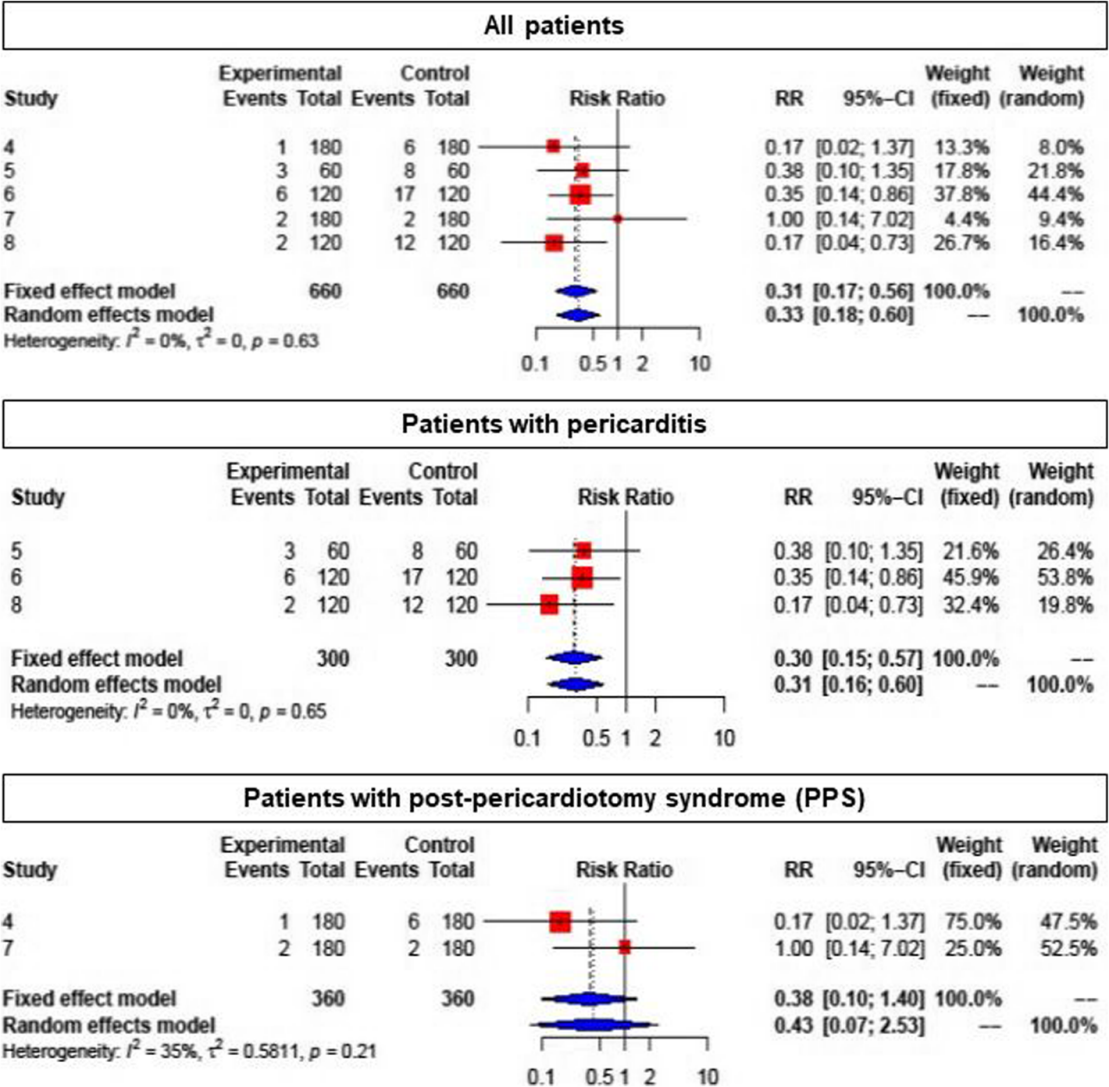
Forest plot graph for the meta-analysis of the rehospitalization rate. Upper graph: all patients, middle graph: pericarditis, bottom graph: post-pericardiotomy syndrome. The mean relative risk (RR) is represented with the blue diamond and the dotted vertical line. The red squares show the RR for the individual studies, the horizontal lines show the corresponding 95% confidence intervals. Experimental: study group treated with colchicine; Control: control group. Events: sum of events, Total: number of patients of the corresponding group, RR: relative risk

Colchicine reduced the need for rehospitalization in five studies (RR: 0.33; 95% CI: 0.18–0.60;). The benefit was significant in patients with pericarditis (RR: 0.31; 95% CI: 0.16–0.60), but not for the patients after heart surgery (RR: 0.43; 95% CI: 0.07–2.53;) (Fig. 6).

### Persistence of symptoms > 72 h

**Fig. 7:**
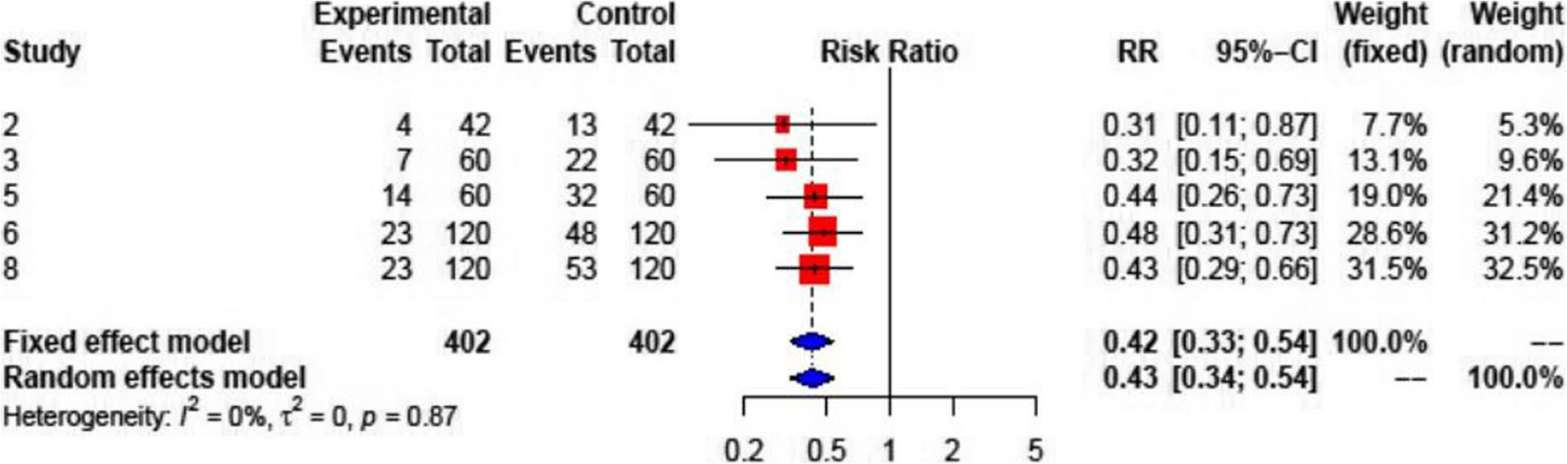
Forest plot graph for the meta-analysis of the symptom persistence after 72 h. The mean relative risk (RR) is represented with the blue diamond and the dotted vertical line. The red squares show the RR for the individual studies, the horizontal lines show the corresponding 95% confidence intervals. Experimental: study group treated with colchicine; Control: control group. Events: sum of events, Total: number of patients of the corresponding group, RR: relative risk

Colchicine reduced the number of patients with persistent symptoms after 72 h in 5 studies with pericarditis patients (RR: 0.43; 95% CI: 0.34–0.54) (Fig. 7).

### Adverse events of colchicine treatment

**Fig. 8:**
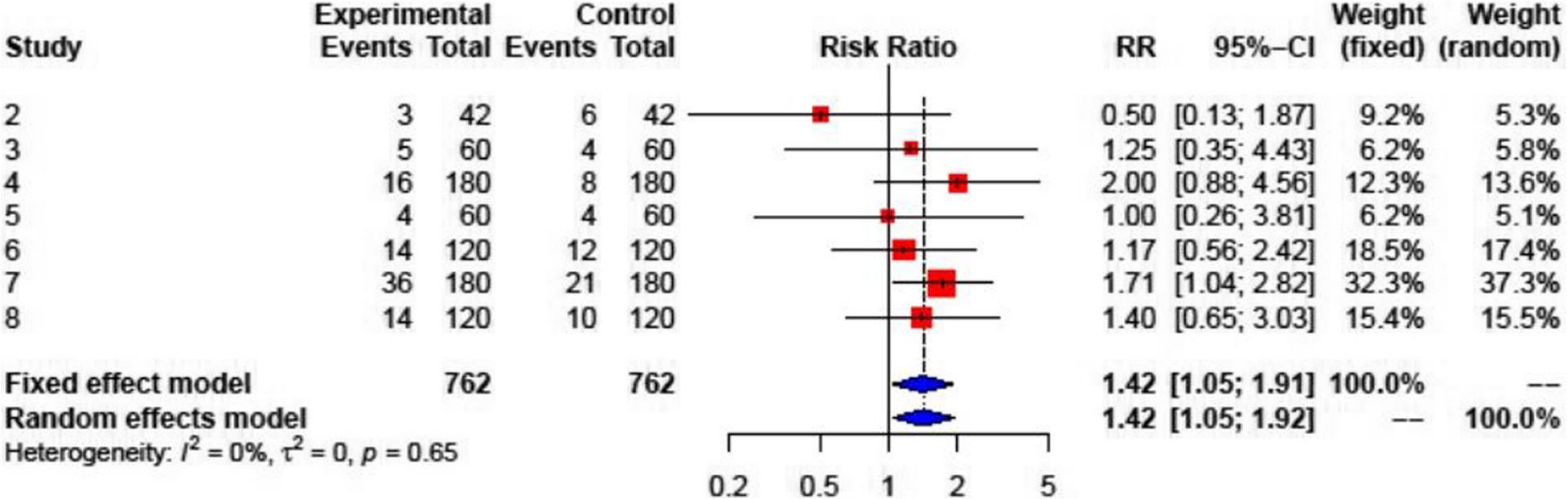
Forest plot graph for the meta-analysis of the adverse events. The mean relative risk (RR) is represented with the blue diamond and the dotted vertical line. The red squares show the RR for the individual studies, the horizontal lines show the corresponding 95% confidence intervals. Experimental: study group treated with colchicine; Control: control group. Events: sum of events, Total: number of patients of the corresponding group, RR: relative risk

Adverse events attributable to colchicine treatment were documented in seven studies, with gastrointestinal intolerance (GI) being the most reported adverse effect of colchicine (RR: 1.42; 95% CI: 1.05–1.92) (Fig. 8). Serious adverse events (SAE) were not reported by any study. The rate of AE ranged from 7% [20] to 20% [11], with gastrointestinal intolerance (GI) being the leading cause of AE, being treated either by direct DW, or by reducing the dosage of colchicine by half, and in cases of GI symptom persistence followed by DW (Table 2). Study #10 did not report detailed AE rates. AE were the main cause of DW, which was confirmed to stop GI. The reported rates of DW ranged from 6.7% [10] to 21.7% [11] (Table 2). Noteworthy, the rates of AE and of DW were not higher in any trial as compared with controls.

## Discussion

Our meta-analysis on 10 studies with 1981 patients treated with colchicine for therapy and prevention of pericarditis or PPS shows that colchicine was effective in reducing the risk of recurrence, the risk of rehospitalization due to pericarditis as well as the number of patients with persistent symptoms after 72 h. Although colchicine is effective in reducing the risk of PPS, as well as the rehospitalization rate after PPS, it was not associated with a significant reduction of postoperative PE in comparison to placebo. Compared to previous related meta-analyses [26–28], our meta-analysis adds to the knowledge on the role of colchicine in the treatment of pericardial disease by including all available RCTs with 1981 patients studied in the treatment groups. The main contribution of our meta-analysis is that, although colchicine is unequivocally effective in the treatment of acute and recurrent pericarditis, as well as for the prevention of PPS, it seems not so efficient in the treatment of patients with postoperative pericardial effusion. Obviously, optimal management of pericardial effusion after cardiac surgery should be investigated in further RCT.

## Anti-inflammatory actions of colchicine

Colchicine is an anti-inflammatory drug used in the treatment of acute and chronic gout, Mediterranean fever and acute arthritis. More than 30 years ago, colchicine was proposed from Rodrigues de la Serna for the prevention of recurrences of acute pericarditis [29]. Unlike NSAID, the anti-inflammatory action of colchicine is not involved in the arachidonic acid pathway. The exact mechanism of anti-inflammatory action is not precisely known. One of the main pathways is the inhibition of the action of leukocytes by causing microtubule depolymerization, thus affecting cell mitosis, chemotaxis, degranulation, phagocytosis and neutrophil motility [30]. Colchicine concentrates preferentially in leukocytes, which enables these anti-inflammatory therapeutic effects at low doses [31].

### Prevention from symptoms and recurrence of PC

Colchicine has been shown to be very effective in reducing recurrence of pericarditis. It had been already included in the first ESC Guidelines for pericardial diseases as a IIa recommendation for the administration together with an NSAID or as monotherapy for the initial attack of pericarditis and prevention of recurrence [1].

Pericarditis recurrence as well as PPS are considered to be due to a localized pericardial autoimmune activation [32, 33]. Detection of increased cytokines and further inflammatory markers in the pericardial fluid, partly with absence of increment of these markers in the blood serum, enhances this concept [34]. Therefore, colchicine can effectively prevent recurrences of pericarditis as well as PPS due to its anti-inflammatory potential. The scenario is different in acute pericarditis, where an infectious pathogenesis seems to be frequently present [35]. In this case, however, colchicine is expected to reduce the recurrence rate, besides enhancing the response of pericarditis to NSAID. It is also evident that colchicine enhances the therapeutic effect of NSAID, thus leading to significantly lower persistence of symptoms after 72 h as well as rehospitalizations due to pericarditis. The latter effect increases the cost-effectiveness of colchicine substantially in comparison to placebo and justifies its use further. In line with these insights, the reported NNT for the prevention of acute or recurrent pericarditis was low, ranging between 3 and 5 [10, 21, 23, 24]. Those effects of colchicine have warranted its inclusion in the latest guidelines as first line therapy for the treatment of acute and recurrent pericarditis [3].

Thus far, the evidence for a differential diagnosis of pericarditis and PE including virological analyses, in accordance to the diagnostic approaches for inflammatory cardiomyopathy [36], may also be important for additional immunomodulatory treatment strategies in PE patients. Whether the different categories of PE diagnosed with complex procedures such as pericardioscopy and epicardial biopsies [37] may have different response patterns for the treatment with colchicine is an issue for further investigations.

### Pericardial effusion after heart surgery

However, our meta-analysis also reveals a significant heterogeneity of the therapeutic benefit of colchicine (Fig. 3; I^2^: 58%; *p* = 0.01). Especially, the publications with the study codes 09 [25] and 10 [18] failed to show significant benefit of colchicine in patients after heart surgery (Fig. 3). This heterogeneity may be largely due to the diversity of study groups (primary and secondary prevention of PC; PPS and PE after heart surgery) included in this meta-analysis. Overall, colchicine did not prove superiority compared to placebo for prevention of PPS in patients after heart surgery (RR: 0.70; 95% CI: 0.48–1.03) (Fig. 5). The NNT reported for PPS prevention was 10 and thus higher compared with the NNT for pericarditis [3–5], and the number needed to harm (NNH) for PPS was 12 [11]. The substantially higher susceptibility of perioperative patients for GI-symptoms may contribute to this imbalance of clinical benefit versus AE and DW this particular patient group.

Most patients (up to 80%) will develop PE to a variable degreeafter cardiac surgery, which is generally mild and rarely results in cardiac tamponade in 1–2% of the post-pericardiotomy cases [38, 39]. Owing to the presumed pericardial inflammation involved in the pathogenesis of PE after cardiac surgery, colchicine could be efficacious in reducing the rate and the size of such pericardial effusions. In the COPPS-2 trial, colchicine administration starting before surgery failed to prevent postoperative pericardial effusion in the whole study cohort but was efficacious in patients with higher CRP [11]. However, this observation is not compatible with the results of the POPE-2 trial, which showed that patients with higher CRP had no decrease of pericardial effusion size [25]. Nevertheless, the pathogenicity of PE after heart surgery is not entirely due to pericardial inflammation, such as in PPS. Hence, colchicine may be able to prevent PPS but not to reduce the rate of clinically relevant PE or to prevent complications of PE after heart surgery that may be due to other causes, such as postoperative bleeding or heart failure. As discussed in [18], one major issue may be the differentiation of the inflammatory pathogenesis of PE after cardiac surgery, since non-inflammatory factors may be prominent in a substantial proportion of patients after cardiac surgery. In patients with PE post cardiac surgery without inflammatory pathogenesis, observational approaches might be the preferred treatment option, since colchicine may be presumably not effective, and potentially harmful due to its known adverse effects, in this substantial subgroup of patients. On the other hand, colchicine was administered late (at the 8th postoperative day) in the POPE-2 study, and at even later (3 weeks after heart surgery) in study #10 [18]. These two trials focused on patients with PE documented with echocardiography after 1–3 weeks after heart surgery, and thus obviously dealt with different patient populations as that of the COPPS [22] and COPPS-2 [11] studies. We therefore conclude that the clinical use of colchicine for the setting of PPS and of postoperative PE should be investigated in further multicenter RCT.

### Adverse effects

Colchicine is associated with adverse effects in up to 10–15% of the patients that may lead to discontinuation of treatment in some patients [20, 21, 32]. The frequent gastrointestinal complaints (mainly diarrhea) may limit colchicine use, but reduction of the dosis will mostly lead to relief of the complaints, and is reversible after DW of colchicine [20, 21]. Although the overall safety of colchicine is unequivocal, use of weight-adjusted doses may decrease [40]. Furthermore, it should be highlighted that several colchicine studies have excluded patients with tuberculous, neoplastic, or purulent causes of PE, hepatic (transaminases 1.5 times the upper normal limit) or renal dysfunction (serum creatinine above 2.5 mg/dL), myopathy or current serum creatine kinase above the upper normal limit, known allergy to colchicine, blood dyscrasias with anemia, leukopenia or thrombocytopenia, and pregnant and lactating women or women of childbearing potential not protected by a contraception method [20, 21, 23]. Noteworthy, the overall rate of AE, including GI, as well as of DW due to AE were not significantly lower in placebo patients as compared with colchicine treated patients in several RCT [10, 11, 20, 21, 23, 24].

## Limitations of the study

The known general limitations of systematic reviews are also generally applicable to this scientific work. Despite the comprehensive and standardized literature search, publication bias may still be relevant in meta-analyses. We only included studies written in English or German language, which might have had an impact on our findings. Diversity of details of the diverse study protocols of the included publications may have had an impact on the overall results of the meta-analysis, which might ultimately have contributed also to the calculated heterogeneity in prevention of PE in PC and PPS patients (see Fig. 3).

## Conclusions

Colchicine is efficient in reducing recurrence of acute and recurrent pericarditis as well as reducing symptom duration and rehospitalization rate in pericarditis and PPS. Mostly observed adverse effects comprise gastrointestinal symptoms that do not compromise treatment safety substantially. Although PPS is effectively prevented by preoperative administration of colchicine, late postoperative administration of colchicine in patients with pericardial effusion (PE) does not seem to reduce the quantity of postoperative PE. The clinical use of colchicine for the setting of PPS and of postoperative PE should be investigated in further multicenter RCT.

## Data Availability

All data are presented within the manuscript.

## Abbreviations

AE: Adverse event(s)
ASA: Acetylsalicylate acid
CI: Confidence interval
DW: Drug withdrawal
GI: Gastrointestinal intolerance
NNH: Number needed to harm
NNT: Number needed to treat
NSAID: Non-steroidal anti-inflammatory drugs
PC: Pericarditis
PE: Pericardial effusion
PPS: Post-pericardiotomy syndrome
RCT: Randomized controlled trial(s)
SAE: Serious adverse event(s)
