# Correction to: Meta-analysis for the value of colchicine for the therapy of pericarditis and of postpericardiotomy syndrome

**DOI:** 10.1186/s12872-019-1195-z

**Published:** 2019-10-18

**Authors:** Leon L. Lutschinger, Angelos G. Rigopoulos, Peter Schlattmann, Marios Matiakis, Daniel Sedding, Paul Christian Schulze, Michel Noutsias

**Affiliations:** 1 Department of Internal Medicine I, Division of Cardiology, Pneumology, Angiology and Intensive Medical Care, University Hospital Jena, Friedrich-Schiller-University Jena, Jena, Germany; 2 Mid-German Heart Center, Department of Internal Medicine III (KIM III), Division of Cardiology, Angiology and Intensive Medical Care, University Hospital Halle, Martin-Luther-University Halle-Wittenberg, Ernst-Grube-Strasse 40, D-06120 Halle (Saale), Germany; 3 Institute of Medical Statistics, Informatics and Data Science (IMSID), University Hospital Jena, Friedrich-Schiller University Jena, Jena, Germany

**Correction to: BMC Cardiovasc Disord (2019) 19:207**

**https://doi.org/10.1186** ***/*** **s12872** ***-*** **019-1190-4**

After publication of the original article [1], we were notified that an author’s name is not complete.

Christian Schulze’s full name is Paul Christian Schulze.

The original article has been corrected.
